# Spatial and Single-Cell Transcriptional Profiling Identifies Functionally Distinct Human Dermal Fibroblast Subpopulations

**DOI:** 10.1016/j.jid.2018.01.016

**Published:** 2018-04

**Authors:** Christina Philippeos, Stephanie B. Telerman, Bénédicte Oulès, Angela O. Pisco, Tanya J. Shaw, Raul Elgueta, Giovanna Lombardi, Ryan R. Driskell, Mark Soldin, Magnus D. Lynch, Fiona M. Watt

**Affiliations:** 1 King’s College London Centre for Stem Cells and Regenerative Medicine, Guy’s Hospital, Great Maze Pond, London, UK; 2 King’s College London Centre for Molecular and Cellular Biology of Inflammation, London, UK; 3 King’s College London MRC Centre for Transplantation, Guy’s Hospital, Great Maze Pond, London, UK; 4 School of Molecular Medicine, College of Veterinary Medicine, Washington State University, Pullman, Washington, USA; 5 Department of Plastic and Reconstructive Surgery, St. George’s National Health Service Trust, London, UK; 6 St. John’s Institute of Dermatology, Tower Wing, Guy’s Hospital, Great Maze Pond, London, UK

**Keywords:** DED, decellularized human dermis, ECM, extracellular matrix, lin^–^, lineage negative, PBS, phosphate buffered saline, qPCR, quantitative PCR, SD, standard deviation

## Abstract

Previous studies have shown that mouse dermis is composed of functionally distinct fibroblast lineages. To explore the extent of fibroblast heterogeneity in human skin, we used a combination of comparative spatial transcriptional profiling of human and mouse dermis and single-cell transcriptional profiling of human dermal fibroblasts. We show that there are at least four distinct fibroblast populations in adult human skin, not all of which are spatially segregated. We define markers permitting their isolation and show that although marker expression is lost in culture, different fibroblast subpopulations retain distinct functionality in terms of Wnt signaling, responsiveness to IFN-γ, and ability to support human epidermal reconstitution when introduced into decellularized dermis. These findings suggest that ex vivo expansion or in vivo ablation of specific fibroblast subpopulations may have therapeutic applications in wound healing and diseases characterized by excessive fibrosis.

## Introduction

Although cell lineage relationships within the epidermis have been studied in detail (Kretzschmar and Watt, 2012), the functional identity of fibroblasts in the dermis is less well characterized (Driskell and Watt, 2015). Fibroblasts are cells that synthesize and integrate structural proteins such as collagen and elastin into the extracellular matrix of most mesenchymal tissues (Kalluri and Zeisberg, 2006, Parsonage et al., 2005, Tomasek et al., 2002, Watt and Fujiwara, 2011). The dermis has distinct layers that are readily identified histologically: the papillary dermis lies closest to the epidermis, whereas the underlying reticular dermis is thicker and contains the bulk of the fibrillar extracellular matrix (Harper and Grove, 1979). Beneath the reticular dermis lies the hypodermis, or dermal white adipose tissue (Driskell et al., 2014, Festa et al., 2011). Other fibroblast subpopulations that have been identified in mouse and human dermis include the dermal papilla cells at the base of the hair follicle (Lee and Tumbar, 2012, Sennett and Rendl, 2012), the cells of the arrector pili muscle, and pericytes that are in close association with blood vessels (Paquet-Fifield et al., 2009).

It has long been suspected that papillary and reticular fibroblasts have distinct identities (Harper and Grove, 1979). In the case of mouse dermis, we and others have shown, via lineage tracing under homeostatic conditions, during wound healing and in skin reconstitution assays, that the papillary and reticular fibroblasts represent functionally distinct lineages that arise from a multipotent progenitor population during embryonic development (Driskell et al., 2013, Rinkevich et al., 2015). In mouse models, papillary fibroblasts are required for new hair follicle formation in the wound bed and in skin reconstitution assays. In contrast, reticular fibroblasts are the first to enter a wound and express the so-called fibroblast activation marker α-smooth muscle actin.

The presence of functionally distinct fibroblast subpopulations within human dermis is potentially of great interest. In addition to the response to wounding, they may play different roles in inflammatory diseases, fibrosis, and skin aging and may offer targetable pathways for the modulation of these pathological states. Previous studies of the human dermis have mechanically separated papillary from reticular dermis with a dermatome. Analysis of explant cultures derived from these separated regions has shown differences in fibroblast behavior in culture and in gene expression (Janson et al., 2012, Lee and Cho, 2005, Sorrell et al., 2004). However, the markers that have been used to distinguish fibroblasts cultured separately from the papillary and reticular dermis (Janson et al., 2012) have not allowed isolation of fibroblast subpopulations directly from the skin.

Here, using a combination of transcriptional profiling of flow-sorted mouse fibroblast subpopulations, spatial profiling of microdissected human dermis, and single-cell RNA sequencing of primary human dermal fibroblasts, we define markers that permit the prospective isolation of human dermal fibroblast subpopulations and show that prospectively isolated fibroblasts from the upper and lower dermis exhibit distinct properties in culture.

## Results

### Differential expression of genes associated with Wnt, extracellular matrix (ECM), and immune signaling in neonatal mouse fibroblast subpopulations

GFP^+^ fibroblasts isolated from the back skin of PdgfraH2BeGFP reporter mice at postnatal day 2 can be separated by flow cytometry on the basis of expression of cell surface markers CD26, Sca1, and Dlk1 (Driskell et al., 2013). To identify additional markers and signaling pathways that distinguish these cell populations, we performed transcriptomic analysis of flow-sorted postnatal day 2 GFP^+^ fibroblasts (see Supplementary Figure S1 online). Reticular fibroblasts were isolated by gating for Dlk1^+^Sca1^–^ cells. Two separate pre-adipocyte populations are positive for Sca1 and can be distinguished from one another using Dlk1:Dlk1^+^Sca1^+^ cells and Dlk1^–^Sca1^+^ cells. Papillary cells were isolated as CD26^+^Sca1^–^. RNA was extracted and subjected to Affymetrix (Santa Clara, CA) microarray analysis and quantitative PCR validation of CD26, Sca1, and Dlk1 expression (see Supplementary Figure S1b–e; see Materials and Methods sections for data access via Gene Expression Omnibus). Marker expression was confirmed in mRNA isolated from flow-sorted populations (see Supplementary Figure S1c) and in the microarrays (see Supplementary Figure S1d).

Principal component analysis was used to assess the relationships between the different cell populations (see Supplementary Figure S1b). The samples aligned along the PC1 (y) axis according to their differentiated state. The two Sca1^+^ (pre-adipocyte) populations were located closest together in PC1. One of the Dlk1^–^Sca1^+^ samples clustered with the Dlk1^+^Sca1^+^ samples and had higher levels of Dlk1 than the other Dlk1^–^Sca1^+^ samples in the microarray, most likely reflecting a technical failure during flow sorting. This sample was therefore excluded from subsequent analysis. Hierarchical clustering of gene expression levels confirmed that the same cell populations from different mice clustered together (see Supplementary Figure S1e).

**Figure 1:**
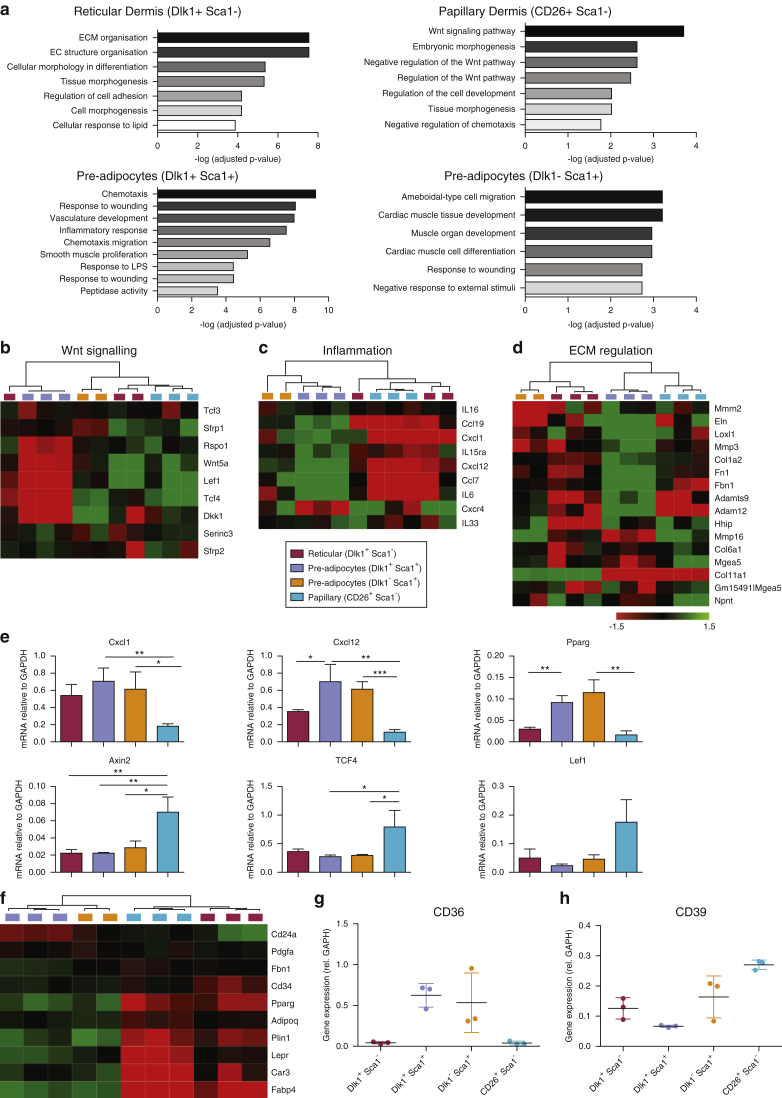
**Differential expression of genes associated with Wnt, ECM, and immune signaling in mouse fibroblast subpopulations.** (**a**) Gene Ontology term analysis of differentially expressed pathways in mouse fibroblast subpopulations. (**b–d**) Heat maps illustrating differential expression (Affymetrix microarray) of genes implicated in (**b**) Wnt signaling, (**c**) inflammation, and (**d**) ECM regulation. (**e**) qPCR validation of selected differentially expressed genes. (**f**) Heatmap comparing expression (Affymetrix microarray) of genes implicated in adipogenesis. (**g, h**) qPCR analysis showing up-regulation of CD36 expression in (**g**) pre-adipocyte populations and (**h**) CD39 in papillary fibroblasts. (**e, g, h**) Gene expression is normalized to GAPDH and expressed as mean ± standard deviation for three biological replicates. ^∗^*P* < 0.05, ^∗∗^*P* < 0.005, ^∗∗∗^*P* < 0.0005. EC, extracellular; ECM, extracellular matrix; qPCR, quantitative PCR.

Gene ontology term analysis of differentially expressed genes (see Figure 1a) confirmed up-regulation of the Wnt signaling pathway in the papillary fibroblasts (Driskell et al., 2013, Rognoni et al., 2016). The top gene ontology term for reticular fibroblasts was ECM organization, whereas the Dlk1^–^Sca1^+^ top gene ontology terms related to muscle, which might reflect the presence of fibro-/adipogenic progenitors in the underlying panniculus carnosus muscle (Natarajan et al., 2010). The top pathways in Dlk1^+^Sca1^+^ cells related to chemotaxis and inflammation.

Heat maps of differentially expressed Wnt, ECM, and inflammation-associated genes are shown in Figure 1b–d. Differential expression of several genes was confirmed by qPCR: Wnt pathway genes Tcf4, Lef1, and Axin 2 were more highly expressed in CD26^+^Sca1^–^ papillary fibroblasts than in the other populations, whereas Cxcl1 and Cxcl12 were significantly down-regulated in papillary fibroblasts (Figure 1e). Dlk1^+^Sca1^+^ cells expressed higher levels of genes encoding fibrillar ECM proteins, such as fibrillin (*FBN1*), than Dlk1^–^Sca1^+^ cells (Figure 1d).

Sca1^+^ mouse dermal fibroblasts can differentiate into adipocytes in vivo and in culture (Driskell et al., 2013, Mastrogiannaki et al., 2016); however, Dlk1^+^Sca1^–^ fibroblasts also have adipogenic activity (Driskell et al., 2013). To gain insights into the nature of the two pre-adipocyte populations, we compared expression of a panel of adipogenic markers (Figure 1f). Both Dlk1^+^Sca1^+^ and Dlk1^–^Sca1^+^ populations expressed higher levels of known pre-adipocyte markers (Rivera-Gonzalez et al., 2016), such as Pparγ and Fabp4, than the other populations (Figure 1f). However, reticular fibroblasts (Dlk1^+^Sca1^–^) had the highest levels of the pre-adipocyte marker CD24 (Festa et al., 2011) (Figure 1f). Both Sca1^+^ populations expressed higher levels of the fatty acid transporter CD36 (Kuniyasu et al., 2003) than the other fibroblast subpopulations (Figure 1g), and CD39 was up-regulated in CD26^+^Sca1^–^ cells (Figure 1h).

### Spatial transcriptional profiling of human dermis

**Figure 2:**
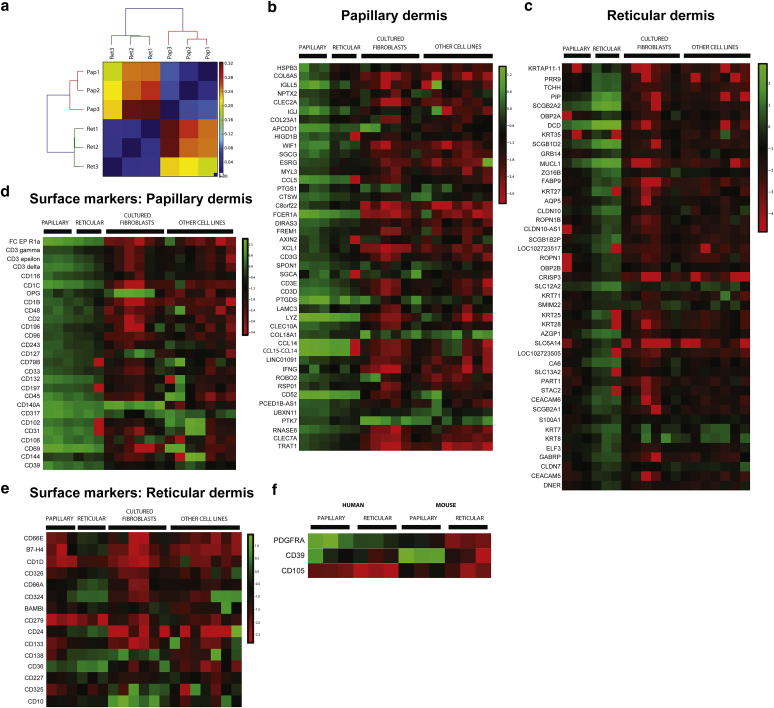
**Spatial profiling of gene expression in human dermis.** (**a**) Hierarchical clustering of gene expression patterns from papillary and reticular dermis in three separate individuals (breast skin samples derived from three donors). Expression was quantified by RNA sequencing (blue, similar; red, dissimilar). (**b**) Genes up-regulated in papillary dermis (>3-fold). (**c**) Genes up-regulated in reticular dermis (>10-fold). (**d**) Cell surface markers for which expression was at least 2-fold up-regulated in papillary dermis. Expression level is indicated separately for each of the three biological replicates (green, high; red, low) and compared with expression in six different cultured fibroblast lines and seven other cultured cell lines. (**e**) Cell surface markers at least 2-fold up-regulated in reticular dermis. (**f**) Cell surface markers up-regulated in papillary dermis that are conserved between human adult dermis and mouse P2 dermis. P2, postnatal day 2.

To test whether spatial differences in gene expression are conserved between mouse and human, we enzymatically removed the epidermis from human skin samples (three adult female breast skin samples from separate individuals) and then microdissected papillary from reticular dermis under a dissecting microscope. RNA was extracted separately from the upper 100 μm (papillary) and 200–500 μm (reticular) human dermal layers and subjected to amplification and RNA sequencing. Hierarchical clustering of gene expression showed that cells in papillary and reticular dermis had distinct gene expression profiles and that samples from the same spatial location derived from different individuals co-associated (Figure 2a).

To identify pan-fibroblast markers (Kalluri and Zeisberg, 2006), we compared our gene expression profiles with published RNA sequencing data from six cultured human fibroblast lines and seven non-fibroblast cell lines (see Supplementary Figure S2a and b online). Pan-fibroblast markers were defined as genes expressed at a high level in both layers of the dermis and all cultured fibroblast lines but not detectable in other cultured cell types. The only cell surface markers meeting these criteria were the known markers CD90 (Saalbach et al., 1998) (although CD90 is also expressed in human embryonic stem cells), PDGFRα, and PDGFRβ (Driskell and Watt, 2015) (see Supplementary Figure S2b). However, the analysis did identify the small leucine-rich proteoglycans LUM and DCN as secreted pan-fibroblast markers (see Supplementary Figure S2a).

Next, we analyzed the genes that were differentially expressed in the different layers of human dermis to find markers for papillary and reticular fibroblasts (Figure 2b and c). One of the most highly enriched markers in papillary dermis was the α5 chain of collagen VI (*COL6A5*) (Fitzgerald et al., 2008, Fitzgerald et al., 2013, Gara et al., 2008). *COL23A1* was also overexpressed in the papillary versus reticular dermis. There was also increased expression of components of the Wnt pathway (*WIF1*, *APCDD1, RSP01, AXIN2*) in papillary dermis, suggesting evolutionary conservation between mouse and human (cf. Figure 1b). The lower dermis did not have a characteristic ECM signature; however, several members of the secretoglobin superfamily (*SCGB2A2, SCGB1D2, SLC12A2*) were highly expressed. Hair follicle-specific genes (*KRTAP11-1, TCHH*) were among the most strongly enriched in the reticular dermis, which is likely due to the lower hair follicle remaining embedded within the dermis when the epidermis was removed before RNA isolation. High expression of the breast epithelial marker *MUCL1* was also a feature of the lower dermis, indicating residual mammary epithelial cells within the preparation.

For functional studies, cell surface markers that distinguish fibroblast subpopulations are very valuable. We therefore filtered the list of differentially expressed genes to identify cell surface markers enriched in papillary (Figure 2d) and reticular (Figure 2e) human dermis. Although CD3γ, CD3δ, and CD3ε were significantly enriched in papillary dermis, this most likely reflected differences in the content of T cells rather than fibroblast subpopulations. We also identified cell surface markers that were differentially expressed in both mouse and human dermal lineages (Figure 2f). No conserved markers of reticular lineages were identified; however, CD39 was identified as a conserved marker of papillary dermal lineages in both mouse and humans.

**Figure 3:**
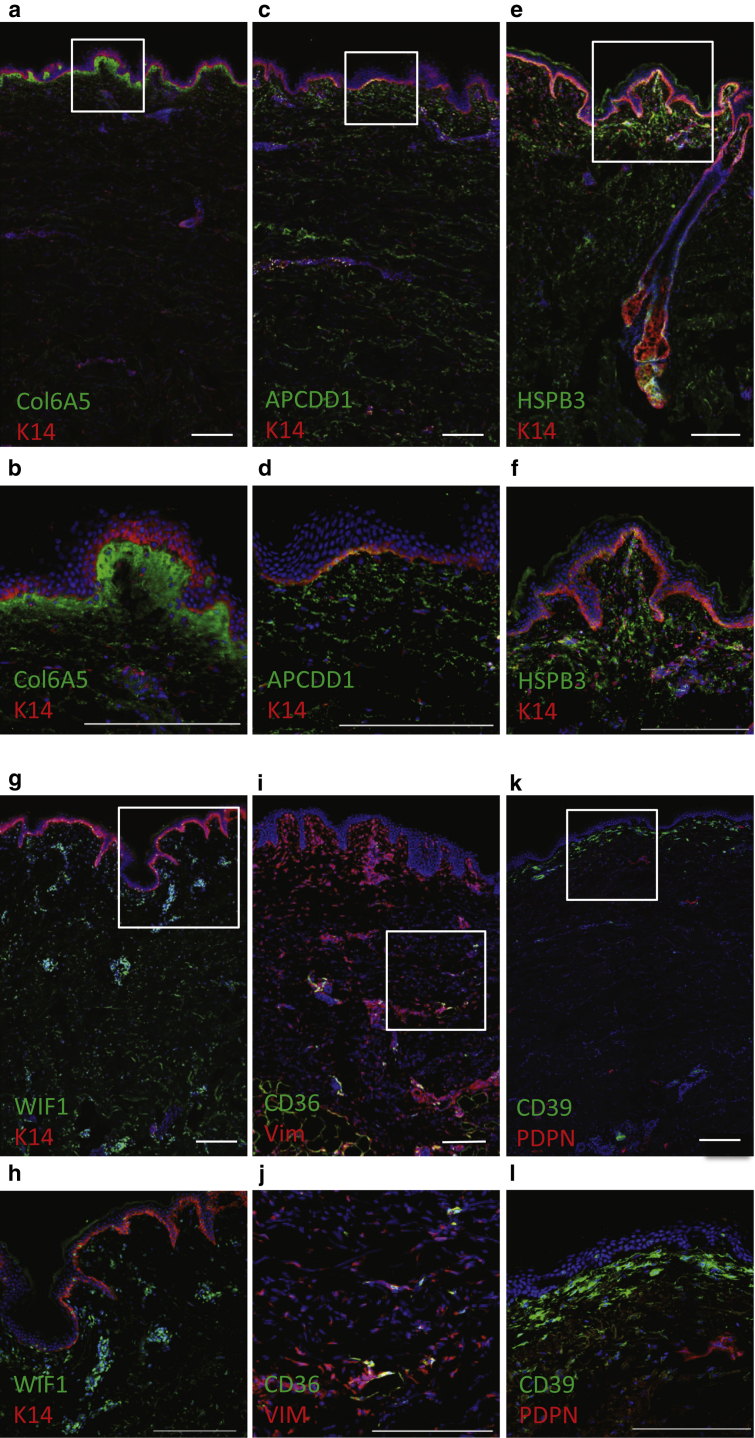
**Immunofluorescence labeling of human dermis with antibodies to candidate fibroblast subpopulation markers identified by spatial transcriptomics.** (**a, b**) Expression of COL6A5 is restricted to the papillary dermis (female breast skin, donor age 22 years). The basal layer of the epidermis is labeled with anti-K14 (COL6A5, green; K14, red). (**c, d**) Expression of APCDD1 is enriched in the papillary dermis (APCDD1, green; K14, red; female back skin, donor age 44 years). (**e, f**) Expression of HSPB3 is enriched in the papillary dermis (HSPB3, green; K14, red; female breast skin, donor age 22 years). (**g, h**) Expression of WIF1 is enriched in vascular structures that are more prominent in the upper dermis (WIF1, green; K14, red; female abdominal skin, donor age 27 years). (**i, j**) Expression of CD36 is highly enriched in the lower dermis (female abdominal skin, donor age 44 years). (**k, l**) CD39 is enriched in the papillary dermis (CD39, green; podoplanin, red; female abdominal skin, donor age 43 years). Scale bars = 200 μm. K14, keratin.

To validate differential expression of the genes identified by RNA sequencing, we performed antibody labeling on skin sections derived from three individuals (breast skin). We confirmed that COL6A5 expression was restricted to papillary dermal fibroblasts (Figure 3a and b) (Martinelli-Boneschi et al., 2017, Sabatelli et al., 2011). Immunostaining for APCDD1 (Figure 3c and d), HSPB3 (Figure 3e and f), and WIF1 (Figure 3g and h) confirmed differential expression of these markers in papillary dermis (Figure 2b). Consistent with their expression in mouse fibroblast subpopulations (Figure 1g and h), CD36 was up-regulated in the lower reticular dermis and hypodermis (Figure 3i and j, data not shown), and CD39 was up-regulated in the papillary dermis (Figure 3k and l). This is in keeping with the in vitro expression of CD36 by adipocyte progenitors and mature adipocytes in vitro (Gao et al., 2017).

### Functional heterogeneity of flow-sorted human fibroblasts

**Figure 4:**
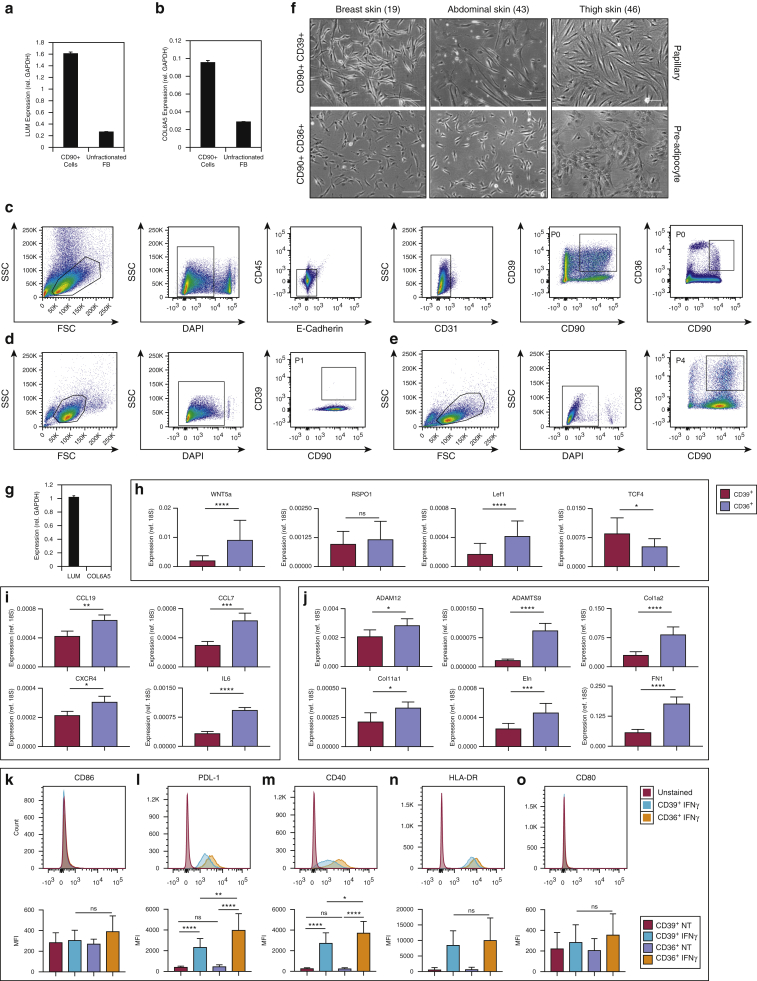
**Human dermal fibroblast subpopulations maintain functional differences in vitro.** (**a, b**) Expression of LUM and COL6A5 is enriched in CD90^+^ population compared with an unfractionated dermal cell suspension. Gene expression normalized to GAPDH and expressed as mean ± standard deviation for three replicates. (**c**) CD39 expression is detectable in primary CD31^–^CD45^–^ECad^–^ cells but is lost after a single passage in culture (**d**). However, expression of (**d, e**) CD90 and (**e**) CD36 is retained. (**f**) lin^–^CD90^+^CD39^+^ and lin^–^CD90^+^CD36^+^ cells exhibit morphological differences in vitro, there is considerable intersample variation. Breast skin donor age, 19 years; abdominal skin donor age, 43 years; and thigh skin donor age, 46 years. Scale bars = 50 μm). (**g**) Quantitative PCR showing retention of LUM and loss of COL6A5 in culture. Gene expression normalized to GAPDH and expressed as mean ± standard deviation for three biological replicates. (**h–j**) Expression of genes associated with (**h**) Wnt signaling, (**i**) inflammation and immunity, and (**j**) ECM remodeling. Gene expression is normalized to 18S and is expressed as mean ± standard deviation for three biological replicates derived from female (age 42 years) abdominal skin, female (age 46 years) thigh skin, and female (age 64 years) abdominal skin. (**k–o**) Modulation of expression of cell surface markers in response to IFN-γ stimulation in culture (blue, CD39^+^IFN-γ; yellow, CD36^+^IFN-γ; red, unstained control). Top row: representative flow plots. Bottom row: quantitation of data from three independent experiments derived from female (age 42 years) abdominal skin, female (age 46 years) thigh skin, and female (age 64 years) abdominal skin. ^∗^*P* < 0.05, ^∗∗^*P* < 0.005, ^∗∗∗^*P* < 0.0005, ^∗∗∗∗^*P* < 0.0001. ECM, extracellular matrix; FSC, forward scatter detector; lin^–^, lineage negative; MFI, mean fluorescence intensity; SSC, side scatter detector.

Based on our analysis of mouse and human fibroblasts, we flow sorted human fibroblasts that were linage negative (lin–) (i.e., CD31^–^CD45^–^E-cadherin^–^) CD90^+^CD39^+^ (papillary) or lin^–^CD90^+^CD36^+^ (lower reticular/hypodermal) and compared their properties after expansion in culture for up to four passages (Figure 4). We confirmed that expression of LUM and COL6A5 was enriched in unfractionated CD90^+^ fibroblasts relative to total dermis (Figure 4a and b). After a single passage, expressions of CD39 and COL6A5 were completely lost from prospectively isolated CD31^–^CD45-^–^ECad^–^ cells; however, expression of CD90, LUM, and CD36 was maintained (Figure 4c–e, g). This shows that culture, rather than competition between different fibroblast subpopulations, leads to the loss of fibroblast markers.

Although expression of CD39 and COL6A5 was rapidly lost, lin^–^CD90^+^CD39^+^ and lin^–^CD90^+^CD36^+^ populations exhibited differences in morphology in culture. There was a tendency toward a more spindle-shaped morphology for CD39^+^ cells, with CD36^+^ cells exhibiting a more epithelioid shape (Figure 4f). However, there was considerable variation between donors, with no obvious correlation between age or donor site (Figure 4f). qPCR showed that genes encoding several ECM components (Figure 4j) and inflammatory mediators (Figure 4i) continued to be more highly expressed in CD36^+^ cells. However, the elevated expression of Wnt pathway genes associated with papillary dermis was lost with culture (Figure 4h).

To examine the functional significance of differential expression of inflammatory mediators (Figure 4i), we performed IFNγ stimulation assays (Figure 4k–o). We observed clear differences in the response of lin^–^CD90^+^CD39^+^ (upper dermis) compared with lin^–^CD90^+^CD36^+^ (lower dermis/hypodermis), with a significant reduction in the upregulation of PDL-1 and CD40 in CD39^+^ cells. This is suggestive of an anti-inflammatory phenotype for upper dermal fibroblasts.

**Figure 5:**
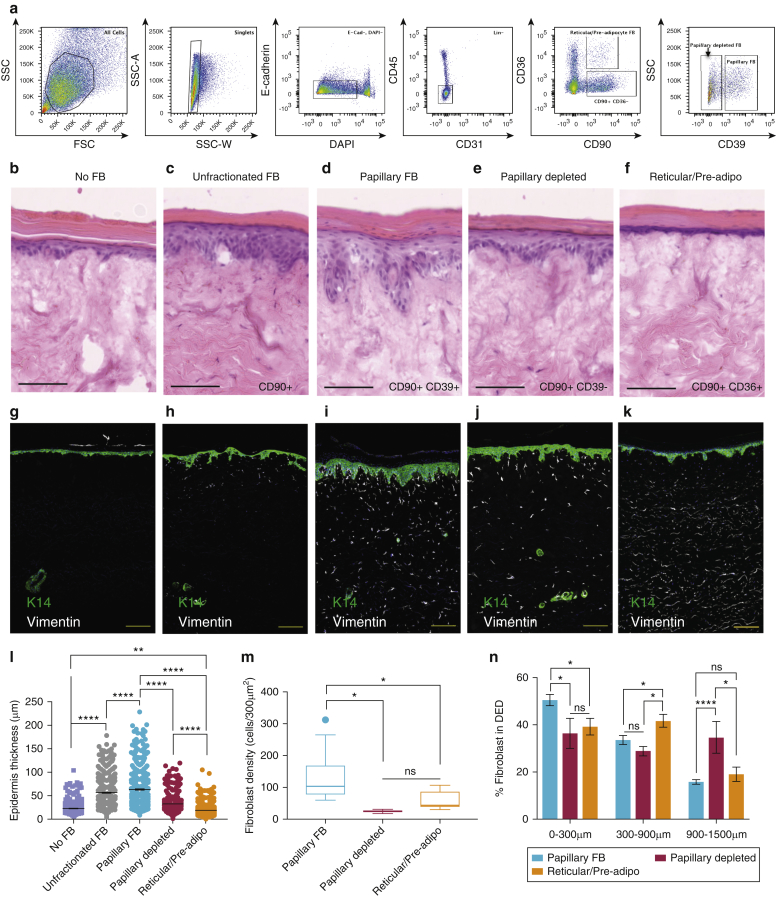
**Comparison of the ability of different fibroblast subpopulations to support epidermal growth on DED.** (**a**) Isolation of fibroblast subpopulations by flow cytometry. (**b–f**) Hematoxylin and eosin staining (scale bars = 100 μm) and (**g–k**) immunofluorescence staining (scale bars = 200 μm) of DED organotypic cultures (**b, g**) without fibroblasts or (**c, h**) seeded with unfractionated (lin^–^CD90^+^) fibroblasts, (**d, i**) CD90^+^CD39^+^ (enriched in papillary) fibroblasts, (**e, j**) CD90^+^CD39^–^ (depleted of papillary) fibroblasts, and (**f, k**) CD90^+^CD36^+^ (reticular/pre-adipocyte) fibroblasts. Keratin 14 (green) marks keratinocytes, and vimentin (white) marks mesenchymal cells (fibroblasts). Experiments were repeated for a minimum of two biological replicates (donor cells derived from female [age 19 years] breast skin and female [age 37 years] breast skin; DED derived from male [age 47 years] abdominal skin, and representative images are displayed. In the case of the CD90^+^CD39^+^ cells, two of three experiments involved additional selection for CD26^–^ cells to further enrich for the papillary cell population (CD90^+^CD39^+^CD26^–^). (**l**) Quantification of epidermal thickness, (**m**) density of fibroblasts within 300 μm of the epidermis, and (**n**) relative abundance of fibroblasts at different depths from the epidermis. adipo, adipocyte; DED, decellularized human dermis; FB, fibroblasts; FSC, forward scatter detector; K14, keratin 14; lin^–^, lineage negative; ns, not significant; SSC, side scatter detector.

Mouse studies have identified a role for reciprocal Wnt signaling between basal keratinocytes and upper dermal fibroblasts in the regulation and maintenance of the epidermal stem cell compartment (Collins et al., 2011, Kretzschmar et al., 2016, Lichtenberger et al., 2016, Rognoni et al., 2016). Although the papillary Wnt gene signature was lost in culture (Figure 4h), it has been previously shown that cultured fibroblasts derived from the papillary dermis support the formation of a normal stratified epidermis in three-dimensional organotypic culture more effectively than fibroblasts from the reticular dermis (Janson et al., 2012, Lee and Cho, 2005, Sorrell et al., 2004). We therefore assessed the ability of cultured fibroblast subpopulations to repopulate decellularized human dermis (DED) and support the formation of an architecturally normal epidermis. Flow-sorted lin^–^CD90^+^CD39^+^, lin^–^CD90^+^CD39^–^, and lin^–^CD90^+^CD36^+^ cells (Figure 5a) were expanded in culture and introduced into the upper surface of DED. Insufficient numbers of cells could be obtained by cell sorting for introduction into DED without the expansion step. Subsequently, primary human keratinocytes were added to the surface of the dermis and cultured at the air-liquid interface.

After 3 weeks in culture, in the absence of fibroblasts, the epidermis had fewer viable cell layers than normal epidermis and lacked the pronounced undulations, rete ridges, characteristic of the healthy tissue (Figure 5b and g). The addition of unfractionated fibroblasts (CD90^+^) led to formation of a thicker epidermis with some rete ridges (Figure 5c and h). lin^–^CD90^+^CD39^+^ cells more effectively supported the formation of a multilayered epithelium (Figure 5d, i, and l) than lin^–^CD90^+^CD39^–^ cells (Figure 5e and j) and lin^–^CD90^+^CD36^+^ cells (Figure 5f and k). After seeding, lin^–^CD90^+^CD39^+^ remained largely restricted to the upper dermis, whereas lin^–^CD90^+^CD39^–^ and lin^–^CD90^+^CD36^+^ cells extended to mid and deep dermis (Figure 5m and n), and fibroblast density was highest in DED samples reconstituted with lin–CD90^+^CD39^+^ cells (Figure 5m).

### Single-cell transcriptional profiling identifies additional human dermal fibroblast subpopulations

**Figure 6:**
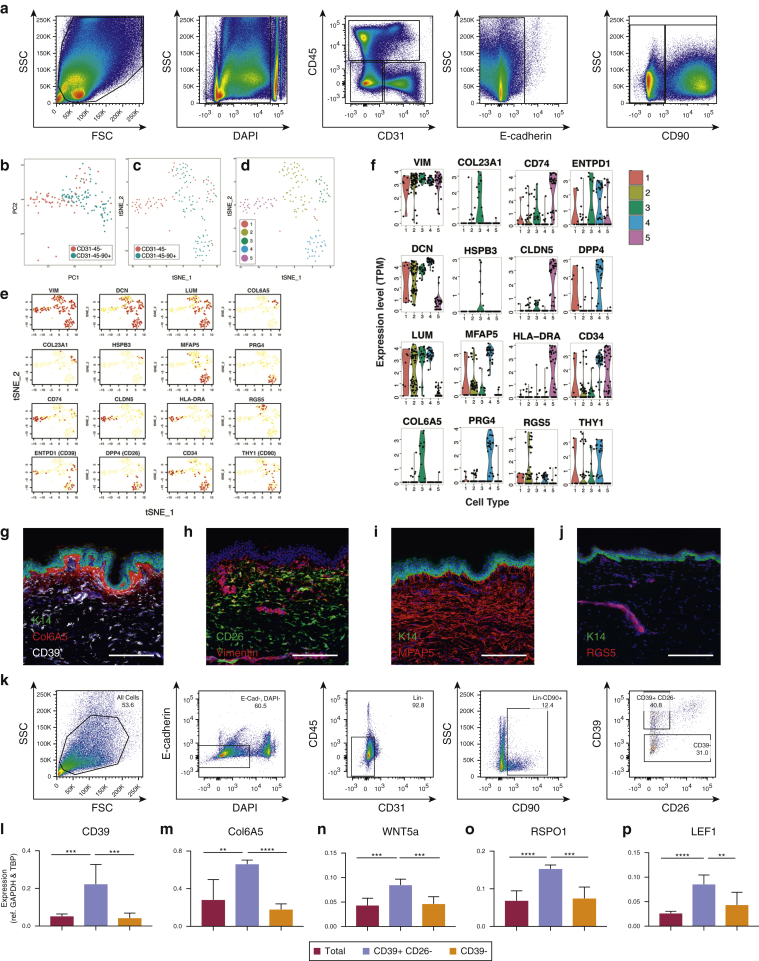
**Single-cell RNA sequencing of human adult dermal fibroblasts.** (**a**) Isolation of lin^–^ cells and lin^–^CD90^+^ cells from human dermis by flow cytometry. Single live cells were isolated from a single donor (female age 64 years, abdominal skin) by gating for forward scatter, side scatter, and DAPI-staining. lin^–^ cells were isolated by gating for CD31^–^CD45^–^ECad^–^. (**b**) PCA analysis of gene expression patterns. (**c, d**) tSNE analysis of gene expression patterns (red, lin^–^; blue, lin^–^CD90^+^). (**d**) Automated clustering of tSNE analysis identifies five dermal fibroblast subpopulations. (**e**) Expression patterns of markers differentially expressed in each of five clusters (red, high expression; yellow, low expression. (**f**) Violin plots illustrating differential expression of marker genes in each of five dermal fibroblast subpopulations. (**g–j**) Immunostaining for candidate fibroblast markers in adult human skin: (**g**) COL6A5, female breast skin, donor age 37 years; (**h**) CD26, female breast skin, donor age 62 years; (**i**) MFAP5, female breast skin, donor age 37 years; and (**j**) RGS5, female back skin, donor age 29 years. Scale bars = 200 μm. (**k**) Isolation of lin^–^CD39^+^CD26^–^ and CD39^–^ dermal fibroblasts by flow cytometry. (**l–p**) Expression of (**l**) CD39, (**m**) COL6A5, (**n**) WNT5A, (**o**) RSP01, and (**p**) LEF1 in lin^–^CD39^+^CD26^–^ and CD39^–^ dermal fibroblasts. Gene expression is normalized to GAPDH and TBP and is expressed as mean ± standard deviation for three biological replicates: male (age 53 years) thigh skin, female (age 52 years) breast skin, and female (age 62 years) breast skin. ^∗^*P* < 0.05, ^∗∗^*P* < 0.005, ^∗∗∗^*P* < 0.0005, ^∗∗∗∗^*P* < 0.0001. FSC, forward scatter detector; K14, keratin 14; lin^–^, lineage negative; PCA, principle component analysis; SSC, side scatter detector; tSNE, t-distributed stochastic neighbor embedding.

Although our analysis of fibroblasts in different layers of human dermis supports the concept that fibroblasts are heterogeneous, it is possible that additional subpopulations are admixed between these two locations. To undertake a more comprehensive assessment of fibroblast subpopulations, we isolated both lin^–^ and lin^–^CD90^+^ cells by flow sorting from human dermis after enzymatic removal of the epidermis (Figure 6a). For five samples of primary human dermis, 12.2% (standard deviation [SD] = 6.7%) of cells were CD90^+^, 9.4% (SD = 4.7%) were lin^–^CD90^+^, and 39.6% (SD = 16.4%) were lin^–^CD90^–^. Thus, fibroblasts constitute a small proportion of dermal cells, most being CD31^+^ endothelial cells (8.1%, SD = 5.2%), CD45^+^ (11.6%, SD = 7.4%) hemopoietic cells and other cell types including sweat glands, neuronal cells, and keratinocytes from hair follicles.

We performed single-cell RNA sequencing for a total of 184 cells derived from the abdominal skin of a single donor (female, age 64 years). To enrich for fibroblasts, half of the cells were flow sorted lin^–^CD90+ cells. However, as we did not wish to potentially exclude unreported, to our knowledge, fibroblast subsets that were CD90^–^, half of the cells were lin^–^CD90^+/–^. t-distributed stochastic neighbor embedding analysis (Figure 6b–d) was performed on global patterns of gene expression for each individual cell; this method groups cells with similar gene expression. Automated clustering of the t-distributed stochastic neighbor embedding analysis (Figure 6d) identified five groups of cells, although group 1 (red) contained only five cells.

To characterize the fibroblast subpopulations, we computationally identified genes that were significantly overexpressed in each group (Figure 6e and f). Vimentin, a mesenchymal cell marker, was expressed in all groups, establishing that no epidermal cells were profiled DCN and LUM—the pan-fibroblast markers we identified by spatial transcriptomics (Figure 2)—were expressed in all groups with the exception of group 5. Group 5 also contained no lin^–^CD90^+^ cells. COL6A5, COL23A1, and HSPB3, identified by spatial transcriptional profiling as localized to the papillary dermis (Figure 2b), were highly enriched in group 3, suggesting that this group represents papillary fibroblasts.

CD74 and HLA-DR4, both components of MHC class II (Karakikes et al., 2012), and CLDN5, a component of tight junctions (Findley and Koval, 2009), marked group 5. Although these cells were vimentin^+^ and lin^–^, the lack of DCN and LUM and expression of CD74 (macrophage inhibitory factor receptor) and HLA-DR indicate characteristics of macrophages/dendritic cells (Brocks et al., 2017, Karakikes et al., 2012). However, CLDN5, another marker of group 5 cells, is an endothelial marker (Kluger et al., 2013). Expression of *Pparγ (PPARG*), and to a lesser extent CD36, both pre-adipocyte markers, was also enriched in group 5 (see Supplementary Figure S3 online), suggesting that pre-adipocytes cluster within group 5, of which they make up a small fraction. Furthermore, a subpopulation of group 5 cells express TEK (TIE2) and might correspond to TIE2-expressing monocyte/macrophages (Patel et al., 2013).

Although group 2 was one of the largest cell clusters, there were few specific markers. One of those, RGS5, is well characterized as a marker of pericytes (Bondjers et al., 2003), and antibodies to RGS5 labeled blood vessel-associated pericytes throughout the dermis (Figure 6j). RGS5-positive cells were limited to the upper half of group 2, suggesting that cells in the lower half of group 2 represent an alternative cellular identity.

CD26, MFAP5, and PRG4 were identified as markers of group 4. This population exhibits a CD39^+^ cell surface phenotype. Because they do not express high levels of *COL6A5,* these cells likely correspond to dermal reticular fibroblasts. CD26 was also a marker of group 1 cells, whereas MFAP5 was expressed by cells in group 2. Antibody labeling indicated that, in contrast to neonatal mouse dermis (see Supplementary Figure S1), cells expressing CD26 were absent from adult papillary human dermis (Figure 6h). MFAP5 was expressed throughout the dermis (Figure 4i). By flow sorting papillary cells on the basis that they were CD39^+^CD26^–^ (Figure 6k and l), we could enrich for expression of the papillary markers COL6A5, WNT5A, RSPO1, and LEF1 (Figure 6m–p). The differences in marker distribution within the dermis were confirmed by staining and flow cytometry of cells from abdomen, back, thigh, and arm skin (data not shown). Thus, fibroblast identity is not restricted by spatial compartmentalization within the dermis.

## Discussion

Our results support the concept (Driskell et al., 2013, Rinkevich et al., 2015) that fibroblasts represent a family of related cell types with specialized functions in the synthesis and maintenance of extracellular matrix and the coordination and regulation of neighboring cell types. Through a combination of spatial and single-cell transcriptional profiling, we have identified at least four human dermal fibroblast subpopulations. The first, which has a cell surface phenotype of lin^–^CD90^+^CD39^+^CD26^–^, is characterized by the expression of specific collagen chains such as COL6A5 and is localized to the upper dermis. The second is lin^–^CD90^+^CD36^+^ cells that are situated in the lower dermis and include pre-adipocytes. The remaining three groups are located throughout the reticular dermis: lin^–^CD90^+^CD39^–^RGS5^+^ cells that correspond to pericytes (Bondjers et al., 2003) and lin^–^CD90^+^CD39^+^CD26^+^ and lin^–^CD90^+^CD39^–^RGS5^–^ cells, which to our knowledge are as yet uncharacterized fibroblast subpopulations. Our single cell analysis is in good agreement with recent studies by Tabib et al. (2018).

In contrast to previous studies of the mouse dermis (Driskell et al., 2009, Driskell et al., 2012, Enshell-Seijffers et al., 2008, Rendl et al., 2005), dermal papilla-associated fibroblasts were not prominent in our dataset, likely reflecting the significantly lower density of hair follicles in human breast and abdomen skin compared with mouse skin. Many of the cell surface markers identified in our previous study of the developing mouse dermis were not conserved in the human. This is not unexpected, because there is poor conservation of cell surface markers for hematopoietic stem cells between humans and mice (Ng et al., 2009). However, all three studies of dermal fibroblast identity have identified CD26 as a key lineage marker, although the subpopulations marked appear to differ. During the early stages of mouse development, CD26 marks an upper dermal lineage; in the adult mouse, CD26 expression is present in a large fraction of the dermis (Rinkevich et al., 2015). Here, we found that CD26 expression, although present in a large fraction of the human adult dermis, is excluded from the uppermost (papillary) fibroblasts. CD26 is a regulator of the inflammatory response (Boonacker and Van Noorden, 2003), and CD26^+^ fibroblasts are reported to contribute to skin fibrosis (Hu and Longaker, 2016).

The specification of fibroblast cellular identity likely reflects a combination of extrinsic signals emanating from the spatial context of the cell and intrinsic mechanisms, including transcriptional and epigenetic regulatory networks. Our results support a key role for signaling via the Wnt pathway in the regulation of dermal fibroblast subpopulation identity. We have found up-regulation of Wnt signaling in both upper dermal mouse and human fibroblast lineages. Wnt signaling is required for normal development of mouse (Ohtola et al., 2008) and human dermis (Grzeschik et al., 2007), and studies have shown a central role for Wnt signaling in hair follicle development and regeneration (Driskell et al., 2012, Kishimoto et al., 2000, Myung et al., 2013, Silva-Vargas et al., 2005). Epidermal Wnt signaling is also implicated in the regulation of the hypodermal adipocyte layer (Donati et al., 2014, Festa et al., 2011) and is critical for maintenance of the epidermis (Watt et al., 2006). We propose that there is a mutually synergistic, Wnt-mediated crosstalk between papillary dermal fibroblasts and basal keratinocytes that is responsible for the maintenance of the cellular identity of the papillary dermal fibroblasts. Loss of this signaling in culture may explain the rapid loss of papillary cell markers.

In addition to differential expression of Wnt pathway components, we observed differential expression of ECM and immunoregulatory genes in subpopulations of mouse and human fibroblasts on isolation from the dermis. This could reflect either a pro-inflammatory state or simply that these subpopulations are primed to respond more readily to injury or infection. We also observed conservation of cell function after in vitro expansion of upper and lower dermal fibroblasts, as evidenced by differences in collagen chain expression and ability to support the normal development of a stratified squamous epithelium in three-dimensional–reconstituted organotypic cell culture. There were also differences in the immunological response to IFNγ stimulation in vitro, and future in vivo investigation of this phenomenon may be informative. Although differences in fibroblast function are conserved in culture, at least for early passages, the expression of key subpopulation markers was rapidly lost. Previous studies have shown that properties of epidermal stem cells displayed in culture or in transplantation experiments differ from those exhibited in vivo (Kretzschmar and Watt, 2012, Watt and Jensen, 2009).

An understanding of the differential contribution of fibroblast subpopulations to human disease may offer strategies for therapy (El-Domyati et al., 2002, Gilchrest, 1996, Gurtner et al., 2008, Jumper et al., 2015, Lee et al., 2004). In this context it could be envisaged that the action of deleterious fibroblast subpopulations could be inhibited, perhaps via inhibition of signaling pathways specific to these subpopulations or via monoclonal antibody-mediated ablation. In mice, experimental depletion of fibroblasts during wound healing can reduce the degree of fibrosis (Dulauroy et al., 2012). Alternatively, beneficial subpopulations can potentially be expanded ex vivo. Fibroblast therapies are already being evaluated to treat poorly healing ulcers (Kirsner et al., 2012) and epidermolysis bullosa (Wong et al., 2008), and papillary fibroblasts appear more effective than other fibroblasts in the construction of tissue-engineered skin substitutes (Sorrell et al., 2008).

## Materials and Methods

### Histology

Surplus surgical waste skin was obtained from consenting patients undergoing plastic surgery. This work was ethically approved by the National Research Ethics Service (UK) (Human Tissue Authority Licence No. 12121, Research Ethics Committee No. 14/NS/1073). For histology, tissue samples were embedded in optimal cutting temperature compound (Life Technologies, Waltham, MA) and stored at –80°C. Sections of 10–16 μm were cut using a Thermo Cryostar Nx70 (Thermo Fisher Scientific, Waltham, MA). Sections were fixed in 4% paraformaldehyde, blocked with a solution of 10% donkey serum, 0.1% fish skin gelatin, 0.1% Triton X-100, and 0.5% Tween-20 (all from Sigma-Aldrich, St. Louis, MO) in phosphate buffered saline (PBS) and labeled with primary antibodies diluted in blocking buffer overnight at 4°C. Sections were washed with PBS and then labeled with secondary antibodies and DAPI for 1 hour at room temperature, washed with PBS, and mounted with Fluorescence Mounting Medium (DAKO, Santa Clara, CA).

### Antibodies

The following primary antibodies were used for immunofluorescence labeling and flow cytometry: anti-mouse CD26 PerCP-Cy5.5 (eBioscience, San Diego, CA; 45-0261-80), anti-human CD26 PE-Cy5 (Biolegend, San Diego, CA; 302708), anti-mouse CD133 PE (eBioscience, 12-1331-80), anti-mouse CD133 APC (eBioscience, 17-1331-81), anti-mouse CD140a APC (eBioscience, 17-1401-81), anti-mouse Ly-6A/E AF700 (eBioscience, 56-5981-82), anti-mouse Dlk1 PE (MBL International/Caltag Medsystems, Buckingham, UK; D187-5), anti-human CD31 PE (eBioscience, 12-0319-41), anti-human CD31 APC-Cy7 (Biolegend, 303119), anti-human CD36 FITC (eBioscience, 11-0369-41), anti-human CD36 PE (Biolegend, 336206), CD39 (eBioscience, 14-0399-82), anti-human CD39 PE (eBioscience, 1112-0399-41), anti-human CD39 APC (Biolegend, 328210), anti-human CD45 AF700 (eBioscience, 111256-9459-41), anti-human CD45 APC-Cy7 (Biolegend, 368516), anti-human CD90 PE (eBioscience, 12-0909-41), anti-human CD90 APC (Biolegend, 328114), anti-human CD324 PerCP/Cy5.5 (Biolegend, 324113), anti-human CD86 PE (Biolegend, 305405), anti-human CD40 APC/Cy7 (Biolegend, 334323), anti-human HLA-DR Pacific Blue (Biolegend, 327016), anti-human CD80 Brilliant Violet 605 (Biolegend, 305225), anti-human CD274 (PD-L1, B7-H1) PE-cyanine7 (eBioscience, 25-5983-41), K14 (Cambridge Bioscience, Cambridge, UK; 906001), PDGFR-α (R&D Systems, Minneapolis, MN; AF307-NA), vimentin (Cell Signaling, Danvers, MA; 5741S), podoplanin (R&D Systems, AF3244), Col6A5 (Abcam, Cambridge, UK; Ab122836), APCDD1 (Abcam, Ab171851), HSPB3 (Abcam, Ab150844), WIF-1 (R&D Systems, MAB134), MFAP5 (Atlas Antibodies HPA010553), and PRG4 (Atlas Antibodies, Bromma, Sweden; HPA028523).

The following secondary antibodies were used: anti-mouse AF555 (Invitrogen, Waltham, MA; A31570), anti-mouse AF647 (Invitrogen, A31571), anti-goat AF488 (Invitrogen, A110550), anti-goat AF555 (Invitrogen, A21432), anti-rabbit AF488 (Invitrogen, A21206), anti-rabbit AF555 (Invitrogen, A31572), anti-rat AF488 (Invitrogen, A21208), anti-rat AF555 (Invitrogen, A21434), and anti-chicken AF488 (Invitrogen, A11039).

### Microscopy

Photomicrographs were taken using a Leica DM IL LED Tissue culture microscope (Wetzlar, Germany). Confocal microscopy was performed with a Nikon A1 Upright Confocal microscope (Tokyo, Japan) using 10× or 20× objectives. Imaging of hematoxylin and eosin-stained sections was performed using a Hamamatsu NanoZoomer slide scanner (Hamamatsu City, Japan). Image processing was performed with Nikon Elements, Image J (Fiji) (National Institutes of Health, Bethesda, MD, Photoshop CS6 (Adobe, San Jose, CA), and Icy software (Quantitative Image Analysis Unit, Institut Pasteur, Paris, France).

### Isolation of neonatal mouse fibroblasts

Postnatal day 2 dermis was harvested as described previously (Jensen et al., 2010). The mouse limbs, tail, and head were removed. An anterior-to-posterior incision in the ventral skin was made, and the skin was separated from the carcass. Skin was incubated for 1 hour at 37°C in a solution of trypsin/EDTA (Sigma-Aldrich) and dispase (Sigma-Aldrich) (50:50), after which the epidermis was peeled off and discarded. The dermis was minced and incubated for 1 hour at 37°C in 0.25% collagenase in FAD basal medium (Gibco, Waltham, MA). The resulting cell suspension was filtered through a 70-μm cell strainer (SLS, Nottingham, England) and centrifuged at 1,800 r.p.m. for 4 minutes at 25°C. The supernatant was removed, and the pellet was washed three times with PBS. Finally, the pellet was resuspended in Amniomax medium (Gibco), and cells were used for flow cytometry and RNA extraction.

### Isolation of adult human fibroblasts

Human adult surgical waste skin was cut into 5-mm–diameter pieces and incubated with dispase for 1 hour at 37°C. The epidermis was peeled off and discarded, and the dermis was digested overnight at 37°C using enzymes from a whole-skin dissociation kit (Miltenyi, Bergisch-Gladbach, Germany). The resulting cell suspension was filtered through a 70-μm cell strainer and centrifuged at 1,500 r.p.m. for 10 minutes at 4°C. The supernatant was removed, and the pellet was washed once with PBS at 1,500 r.p.m. for 4 minutes at 4°C. The pellet was resuspended in PBS plus 1% fetal bovine serum for flow cytometry or lysis buffer containing 2-mercaptoethanol (Qiagen, Hilden, Germany) for RNA extraction.

### Cell culture

Human adult dermal fibroblasts were cultured in DMEM plus 10% (volume/volume) fetal bovine serum, 2 mmol/L l-glutamine, and 100 U/ml penicillin, 100 μg/ml streptomycin (Gibco), or Amniomax C100 medium with Amniomax C100 supplement (Gibco). Culture flasks were incubated at 37°C in a humidified atmosphere with 5% CO_2_ and passaged every 3–5 days, when 80% confluent. Cells were used between passages 1 and 6 for all studies. Stock cultures of primary normal human keratinocytes (strain km) were obtained from surgically discarded foreskin and grown on 3T3-J2 feeder cells. Normal human keratinocytes were used for DED experiments between passages 2 and 5. 3T3-J2 fibroblasts were originally obtained from James Rheinwald (Department of Dermatology, Skin Disease Research Center, Brigham and Women's Hospital, Boston, MA), not authenticated. All cell stocks were routinely tested for mycoplasma contamination, and results were negative. Normal human keratinocytes were cultured in complete FAD medium, containing one part Ham’s F12, three parts DMEM, 10^−4^ mol/L adenine, 10% (volume/volume) fetal bovine serum, 0.5 μg/ml hydrocortisone, 5 μg/ml insulin, 10^−10^ mol/L cholera toxin, and 10 ng/ml EGF, on mitotically inactivated 3T3-J2 cells as described previously (Jones and Watt, 1993, Rheinwald and Green, 1975). For IFN-γ–stimulation assays, human dermal fibroblasts were stimulated with 1,000 U/ml IFN-γ (Sigma-Aldrich) for 72 hours in growth medium before harvesting for analysis by flow cytometry.

DED was prepared as described previously (Rikimaru et al., 1997). Briefly, adult human skin was divided into 1–2 cm^2^ squares and heated at 52°C for 20 minutes, and the epidermis was separated from the dermis with forceps. The dermis was depleted of cells by at least 10 freeze-thaw cycles and irradiated once with 60 Gy. Before fibroblasts were seeded onto DEDs, the tissue was placed into six-well hanging cell culture inserts (Millipore, Billerica, MA) and equilibrated with DMEM. Fibroblasts, 5 × 10^5^ cells/DED, were injected into the DED using U-100 insulin syringes (BD, Franklin Lakes, NJ) from the epidermis surface and then incubated for 72 hours completely submerged in DMEM. Medium was changed to FAD medium, DEDs were placed at the air-liquid interface and 1 × 10^6^ keratinocytes were seeded on top of each DED. DEDs were maintained in culture with FAD medium at the air-liquid interface for 3 weeks with medium changes every 48 hours.

### Flow cytometry

Disaggregated dermal cells were labeled with antibodies in PBS plus 1% fetal bovine serum for 45 minutes at 4°C. DAPI was used to exclude dead cells. Fluorescence minus one controls were used during the experimental set up. After incubation, cells were centrifuged at 1,500 r.p.m. for 4 minutes at 4°C and washed three times in PBS plus 1% fetal bovine serum. Pellets were resuspended in PBS plus 1% FCS and filtered through a 50-μm cell strainer. Data acquisition was performed using the BD FACSCanto II fluidics and LSRFortessa cell analyzer systems. Cell sorting was performed on the BD FACSAria II and BD FACSAria III Fusion cell sorters. For gate setting and compensation, unlabeled, single-labeled cells and compensation beads (BD) were used as controls. Data analysis was performed using FlowJo software, version 10 (Tree Star, Ashland, OR).

### Quantitative real-time reverse transcriptase–PCR

Total RNA was isolated using the Qiagen RNeasy mini kit, and cDNA was synthesized using the QuantiTect Reverse Transcription kit (Qiagen) or Superscript III First-Strand Synthesis (Thermo Fisher). Additional RNAse H treatment was completed at the end of the reaction by adding 1 μl of RNAse H per 30 μl reaction for 20 minutes in PCR block at 37°C.

Quantitative real-time reverse transcriptase–PCR reactions were performed on CFX384 Real-Time System (Bio-Rad, Hercules, CA) using the standard protocols for TaqMan Fast Universal PCR Master Mix with TaqMan probes, or using SYBR-Green Master Mix (Life Technologies) using qPCR primers (published or designed with Primer3). Values were normalized to GAPDH, 18S, or TBP expression levels using the ΔCT method. Each reaction was completed with at least biological triplicates unless otherwise stated. The following TaqMan probes were used: CD36 Mm01135198_m1, AKAP12 Mm00513511_m1, CHODL Mm00507273_m1, mouse GAPDH endogenous control (4352339E), CD39 Mm00515447_m1, Akr1c18 Mm00506289_m1., IL6 Mm00446190_m1, and NRK Mm00479081_m1.

### Affymetrix microarrays

cDNA was fragmentated and labeled with the Affymetrix GeneChip Labeling Kit. The labeled DNA target and fragmented cDNA were hybridized on Mouse Gene 2.0 ST Arrays (Affymetrix). The microarrays were scanned on a GeneChip scanner 3000 (Affymetrix).

The data are deposited in Gene Expression Omnibus (GSE104225).

### RNA sequencing

Spatial RNA sequencing was performed using fresh human skin samples (from three separate individuals). Skin samples were incubated with Dispase II (Stemcell Technologies, Vancouver Canada) for 1 hour at 37°C permitting separation of the epidermis. The dermis was separated into papillary (upper 100 μm) and reticular (200–500 μm) dermis by microdissection under a dissecting microscope. Separated papillary and reticular dermis samples were subsequently transferred to lysis buffer containing 2-mercaptoethanol (PureLink RNA micro scale kit, Invitrogen) and homogenized for 2 minutes using a mechanical homogenizer (Polytron, Kinematica, Luzern, Switzerland). Subsequent RNA extraction was performed using the PureLink micro kit according to the manufacturer’s instructions. Library preparation was performed according to the SmartSeq2 protocol (Picelli et al., 2014).

For single-cell RNA sequencing, single cells were isolated by flow cytometry (as described) from the abdominal skin of a single donor. Cells were sorted into individual wells of a 96-well plate containing 2 μl lysis buffer (0.2% (volume/volume) Triton X-100 and 2 U/μl recombinant RNase inhibitor (Clontech, Mountain View, CA). Library preparation was performed with SmartSeq2, followed by the Nextera XT protocol (Illumina, San Diego, CA). Sequencing was performed on the Illumina Hiseq2000 or Hiseq 25000 with TruSeq SBS v3 chemistry.

Reads were mapped with Tophat2 (Kim et al., 2013). Gene-specific expression was quantified using featureCounts (Liao et al., 2014). The data are deposited in Gene Expression Omnibus (GSE109822). Cells for which less than 200,000 total reads were mapped were excluded from further analysis. Reads were normalized on a per-cell basis to give mapped reads per million. Statistical analysis and visualization of single-cell sequencing data were performed using the Seurat package for R according to the recommended protocol (Satija Lab, New York, NY).

### Graphing and statistical analysis

Graphs were generated using Excel (Microsoft, Redmond, WA) and GraphPad (La Jolla, CA) Prism 6 software. Data are mean ± standard error of the mean. One-way analysis of variance parametric test with Bonferroni posttest or Student tests were performed for experiments, with *P* < 0.05 considered significant.

## ORCID

Christina Philippeos: http://orcid.org/0000-0001-8654-0291

Fiona M Watt: http://orcid.org/0000-0001-9151-5154

## Conflict of Interest

The authors state no conflict of interest. Some of the work described in this manuscript is the subject of a patent application by MDL, CP and FMW.
