# Supplementary figures and images for: Spatial and Single-Cell Transcriptional Profiling Identifies Functionally Distinct Human Dermal Fibroblast Subpopulations
Source: J Invest Dermatol. 2018 Apr;138(4):811–25. doi: 10.1016/j.jid.2018.01.016 (PMC5869055; doi:10.1016/j.jid.2018.01.016)

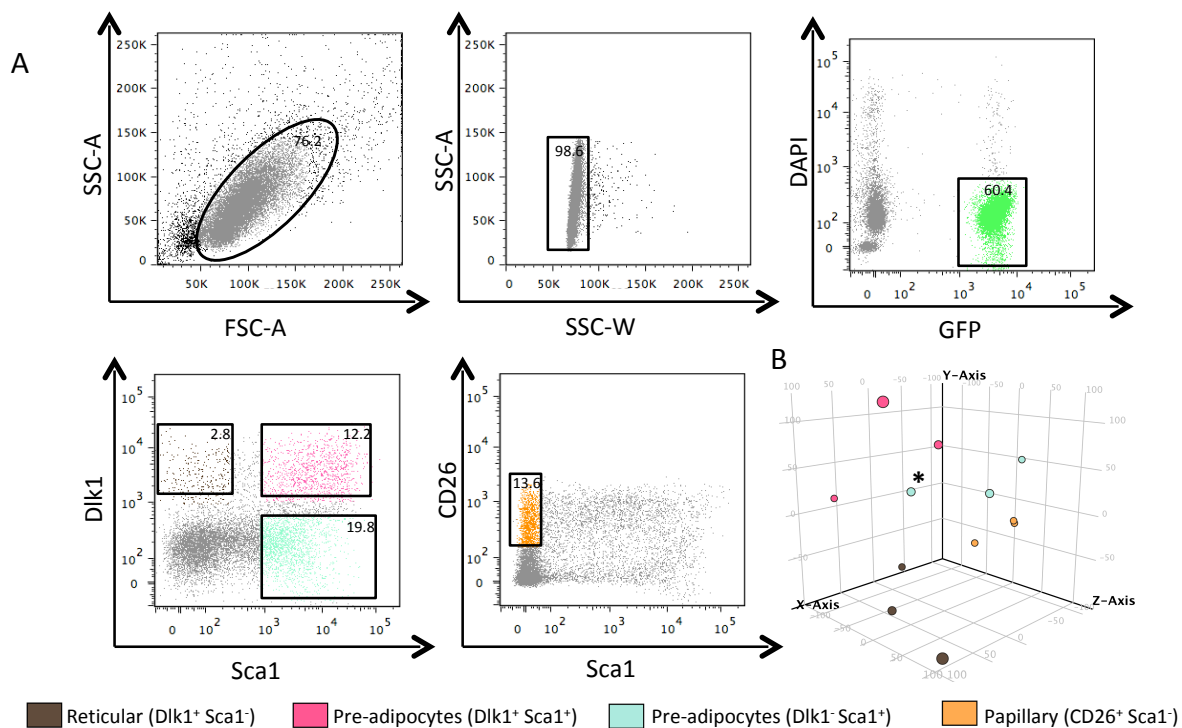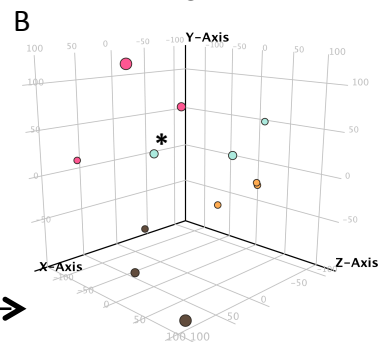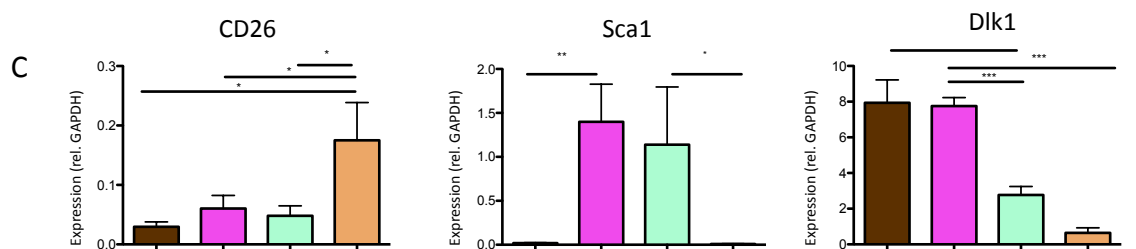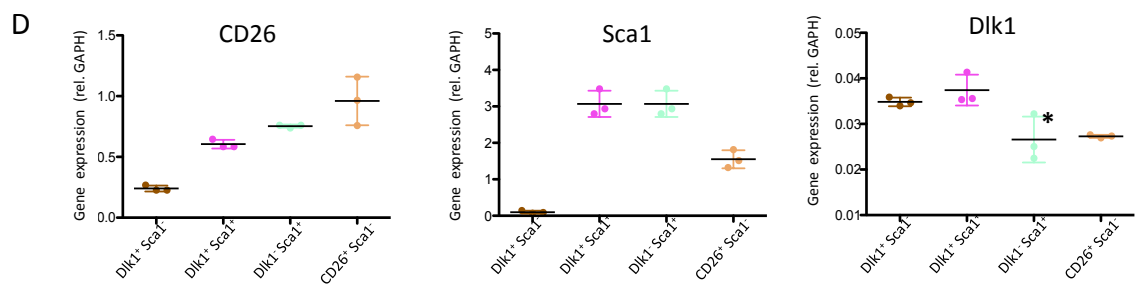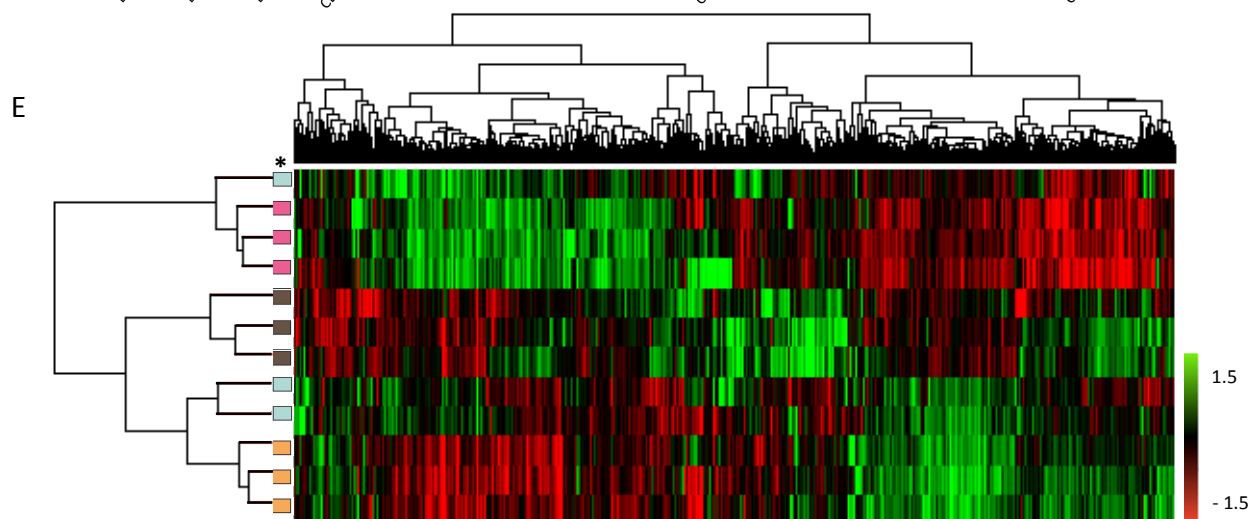

Supplementary Figure S1

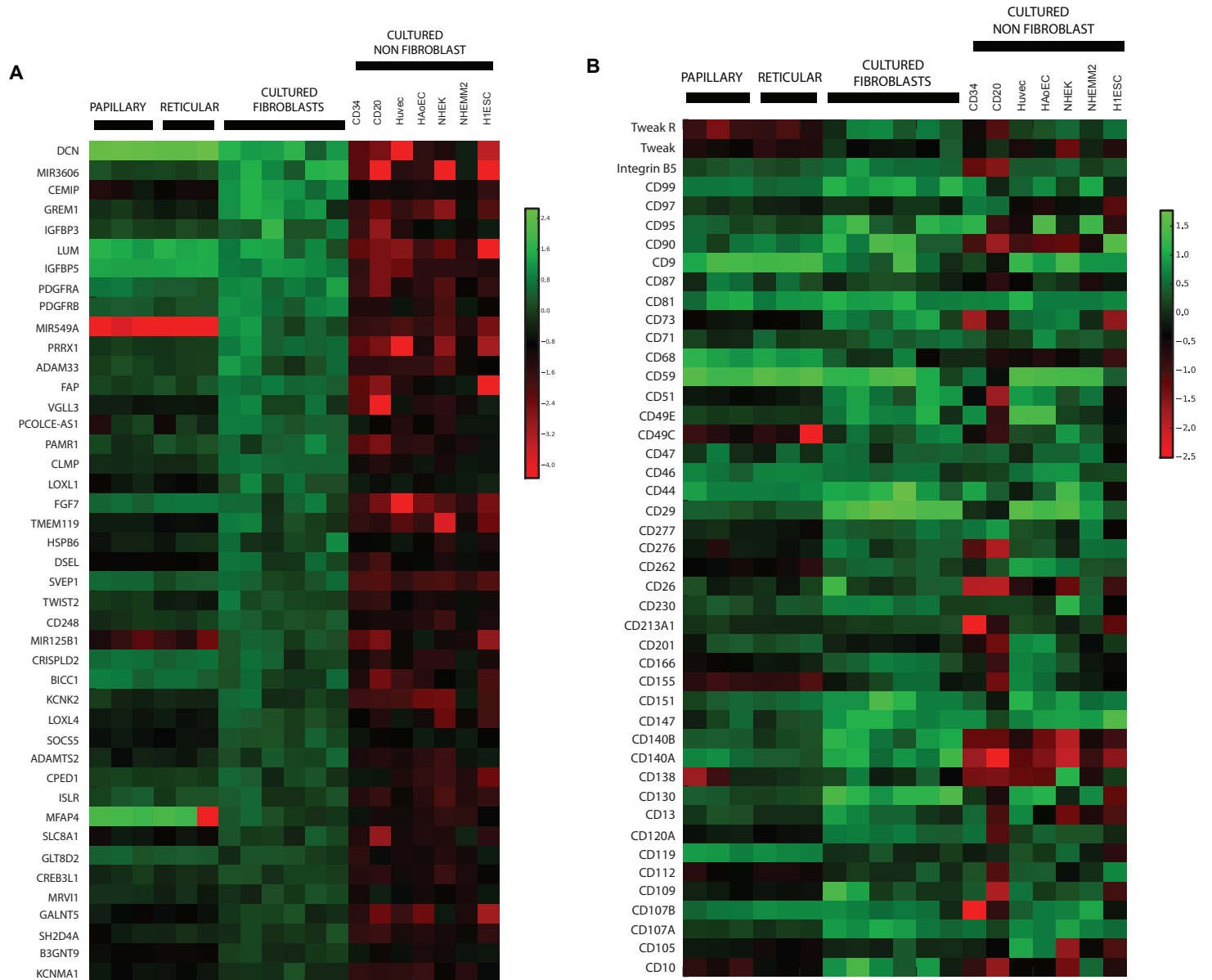

Supplementary Figure S2

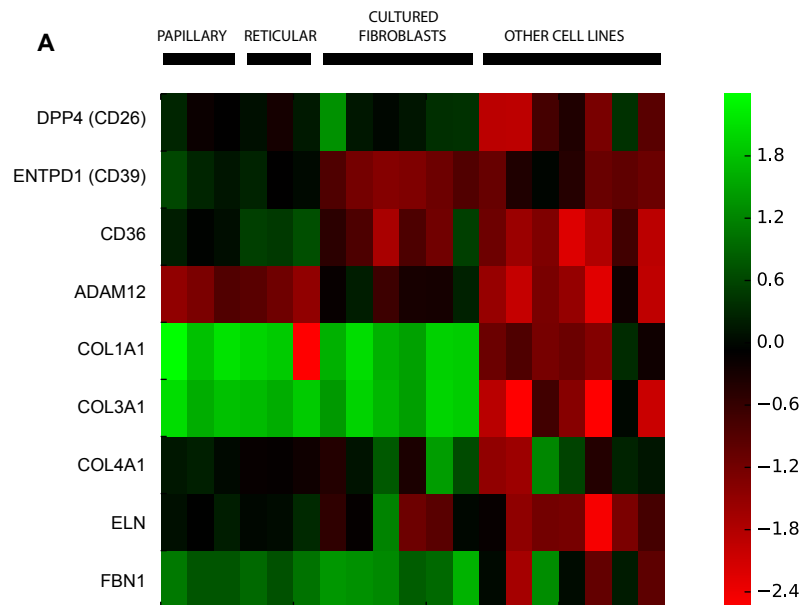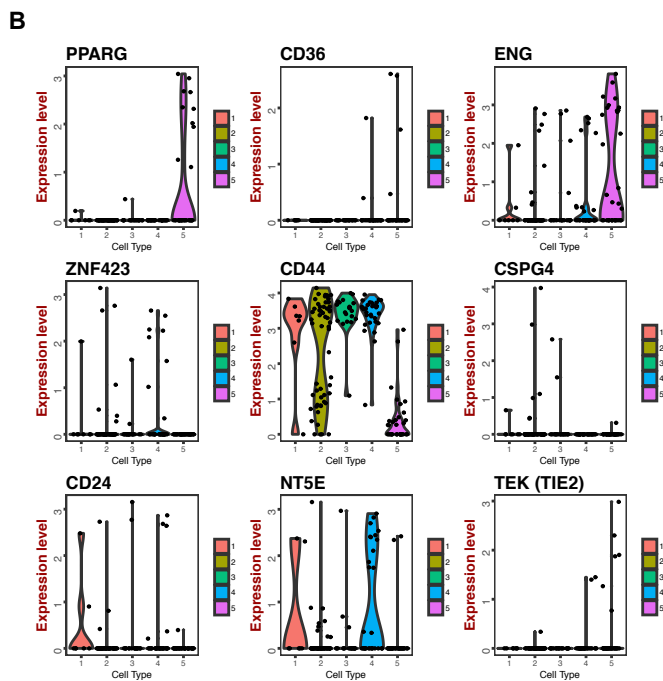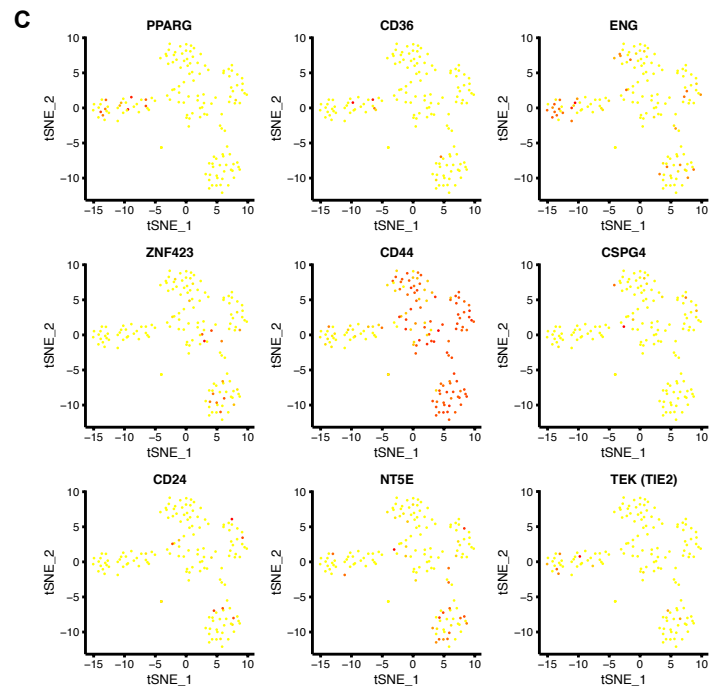

Supplementary Figure S3

Supplement: Supplementary Figures S1–S3 [file mmc1.pdf]
